# Community Water Fluoridation and Birth Outcomes

**DOI:** 10.1001/jamanetworkopen.2025.54686

**Published:** 2026-01-20

**Authors:** Benjamin Krebs, Lisa Simon, Hannes Schwandt, Samantha Burn, Matthew Neidell

**Affiliations:** 1 Faculty of Business and Economics, University of Basel, Basel, Switzerland; 2 Department of Medicine, Brigham and Women’s Hospital, Boston, Massachusetts; 3 Buehler Center for Health Policy and Economics, Northwestern University, Chicago, Illinois; 4 Imperial College Business School, London, United Kingdom; 5 Department of Health Policy and Management, Mailman School of Public Health, Columbia University, New York, New York

## Abstract

**Importance:**

Community water fluoridation (CWF) is a widely implemented public health intervention aimed at preventing dental caries. However, concerns have emerged about potential unintended birth outcomes of fluoride exposure, particularly birth weight, which is a widely accepted summary measure of infant health and has been found to be associated with later-life health and human capital.

**Objective:**

To evaluate the association between prenatal exposure to CWF and birth outcomes.

**Design, Setting, and Participants:**

This cohort study was conducted using an event-study analysis with a difference-in-differences (DID) approach that exploited the staggered rollout of CWF across US counties from January 1968 to December 1988. The study included birth outcomes from singleton births obtained from the National Vital Statistics System’s Natality Detail Files. The analysis compared within-county changes in birth outcomes before and after the introduction of CWF, using counties that never fluoridated or had not yet fluoridated as the control. Data were analyzed between February 4 and October 28, 2025.

**Exposure:**

County-level CWF.

**Main Outcomes and Measures:**

The primary outcome was change in mean birth weight, and secondary outcomes were incidence of low birth weight (<2500 g), gestational length (weeks), and incidence of prematurity (gestational age <37 weeks). Community water fluoridation exposure was measured as the population-weighted share of county residents served by fluoridated water based on Centers for Disease Control and Prevention Fluoridation Census data.

**Results:**

The study sample included 11 479 922 singleton births (mean [SD] gestational age, 39.5 [0.8] weeks; 51.2% boys; mean [SD] birth weight, 3337.4 [172.8] g; highest mean [SD] maternal age proportions, 0.36 [0.13] aged 20-24 years and 0.27 [0.12] aged 25-29 years) across 677 counties (408 CWF treated [60.3%] and 269 [39.7%] never treated) over the 21-year period, aggregated to the county-month level. No evidence of an association of CWF with birth weight or other birth outcomes was found (DID estimate, −0.53; 95% CI, −4.75 to 3.70). Event-study estimates showed no discernible pretreatment trends and no significant changes following CWF adoption, with estimates small in magnitude across all posttreatment periods, ranging from −8.44 g (95% CI, −20.41 to 3.53 g) to 7.20 g (95% CI, −5.45 to 19.85 g). Findings were supported by sensitivity analyses incorporating state-specific time trends, alternative exposure thresholds, and tests for compositional changes in births.

**Conclusions and Relevance:**

This cohort study of more than 11 million births found no association of CWF with adverse birth outcomes. These findings provide reassurance about the safety of CWF during pregnancy and underscore the value of rigorous causal designs in evaluating potential adverse effects of public health interventions.

## Key Points

**Question:**

Is community water fluoridation (CWF) associated with changes in birth weight among infants born before and after its introduction in US counties?

**Findings:**

In this cohort study of 11 479 922 singleton births across 677 counties between 1968 and 1988, aggregated to the county-month level, CWF was not associated with statistically significant changes in birth weight.

**Meaning:**

These findings suggest that concerns about changes in birth weight associated with CWF may be unfounded, underscoring the value of rigorous research designs in evaluating potential adverse effects from CWF.

## Teaser

This cohort study evaluates the association between prenatal exposure to community water fluoridation and birth weight and other birth outcomes in US counties.

## Introduction

Community water fluoridation (CWF) has long been promoted as a safe and effective public health intervention for preventing dental caries. While its benefits have been well documented, recent studies have raised concerns about potential unintended adverse effects, particularly from prenatal and early-life fluoride exposure. Much of this literature has focused on cognitive outcomes, such as IQ, but the findings were often derived from observational studies with limited ability to address confounding.^1,2^ These methodological weaknesses have contributed to uncertainty about whether the associations reported in the literature may be causal or underlie population differences.

In this study, we focused on birth weight as an alternative, though we believe complementary, outcome in assessing the potential adverse effects of fluoride exposure during pregnancy. Although distinct from cognitive outcomes, birth weight is a widely accepted summary measure of infant health and has been associated with later-life health and human capital.^3^ The rapid development of the fetus provides a microcosm of human development that is sensitive to various potential insults. From a methodological perspective, birth weight is advantageous because it reflects the short and well-defined period of exposure from conception to birth, thereby limiting concerns about long-term cumulative exposure and reducing opportunities for bias from unobserved postnatal factors. From a data perspective, birth weight is a consistently measured outcome for the universe of US births over a long period, and birth certificates identify the mother’s county of residence, which allows linkage to CWF exposure.

Emerging biological evidence has supported the plausibility of an association between prenatal fluoride exposure and fetal development. Fluoride crosses the placenta^4^ and has been detected in amniotic fluid and cord blood, indicating direct fetal exposure during gestation. Proposed mechanisms for a negative impact from maternal fluoride exposure have included altered maternal thyroid function, oxidative stress, or disrupted placental nutrient transfer, all of which may influence fetal growth.^5,6^ Several correlational studies have found higher maternal fluoride levels associated with decreases in birth weight.^7,8,9^

To investigate the association between CWF and birth weight, we used an event-study difference-in-differences (DID) design that exploits the staggered rollout of CWF across the US over several decades. Specifically, we compared changes in birth weight before and after the introduction of CWF in treated communities with a control group of counties that either never fluoridated or had not yet fluoridated. By focusing on within-community changes over time and including control communities, our design adjusted for both time-invariant differences across locations and broader temporal trends. This approach is part of a class of statistical methods increasingly used in population health research to limit threats from confounding factors when experimental designs are infeasible.^10,11,12^

## Methods

### Data

This cohort study used data from January 1968 to December 1988, taken from the Centers for Disease Control and Prevention’s 1992 Water Fluoridation Census.^13^ Although earlier CWF data are available, we began our analysis in 1968 to align with the availability of birth data. The fluoridation census provides detailed information on the fluoridation status of every public water system in the US, including the month and year fluoridation began, whether fluoride was naturally occurring or chemically added, the county served, and the population served by each system as of December 1992. The study did not meet the criteria to be considered human participant research per the Common Rule, as there was no interaction or intervention with individuals, and private, identifiable information was not collected. The study followed the Strengthening the Reporting of Observational Studies in Epidemiology (STROBE) reporting guideline.

For each county, we constructed the month and year in which fluoridation was initiated, if ever, following the methodology outlined in Glied and Neidell.^14^ We merged the fluoridation census with population data from the 1990 Census of Population and Housing to calculate the share of the county population served by fluoridated water in 1990. To estimate county-level fluoridation rates in earlier years, we assumed that the share of the population served by each water system remained constant over time. Using the start date of fluoridation, we assigned the estimated share fluoridated to all subsequent months and 0 to all prior months. In counties with multiple fluoridating water systems, we computed a population-weighted average of the fluoridation share across systems. This process yielded a county-month panel of CWF exposure from 1968 to 1988.

Birth outcome data for the same period were from the Natality Detail Files of the National Vital Statistics System maintained by the National Center for Health Statistics. These microdata are drawn from birth certificates filed by all states and the District of Columbia. Prior to 1972, the data represented a 50% sample of births in all states. From 1972 onward, the data have included a mix of 50% samples from some states and 100% from other states, with the number of states reporting 100% slowly expanding until full national coverage by 1985.^15^ The dataset includes demographic and maternal characteristics, such as date of birth, parental age and education, marital status, birth order, infant sex, self-reported race of the mother based on the historical categorization of 1968 (Black, White, other), and geographic identifiers. Race was included as a control variable because it is associated with average birth weight. We excluded observations with multiple births (due to influence on birth weight), missing birth weight, or missing county information. Birth weight data were aggregated to the county-month-year level and merged with the constructed CWF exposure panel.

### Statistical Analysis

Our main approach for estimating the association between CWF and birth outcomes was a staggered-entry DID event-study design. We compared changes in birth outcomes over time in areas that adopted CWF, using never-treated and not-yet–treated areas as the control group. This approach produces estimates of both a series of coefficients prior to CWF adoption as a tool for model diagnostics and a series of coefficients after CWF to explore a dynamic treatment response.^16,17^ In this way, we assessed whether the estimated association between CWF and birth outcomes changed over time.

Our primary outcome was mean county-level birth weight. As secondary outcomes, we evaluated the fraction of births that were low birth weight (<2500 g), gestational length in weeks, and prematurity (gestational age <37 weeks). We selected these outcomes based on prior literature establishing them as measures of newborn health.^3^ Details of the model are provided in the eMethods in Supplement 1.

For comparison purposes, we also produced static, more traditional DID estimates by defining the pretreatment period as 1 year before CWF adoption and the posttreatment period as 9 to 21 months after adoption, which corresponds to the interval in which all newborns were exposed to CWF throughout the entire prenatal period. This approach, described in more detail in the eMethods in Supplement 1, estimated an average treatment response after CWF adoption.^17^ It continued to exploit the staggered rollout of CWF but limited comparison with periods more immediately surrounding CWF adoption.

The event-study DID analysis adhered to the prespecified analysis plan, which was developed to limit the scope for data mining to uncover particular patterns in our data.^18^ The static DID estimates and event study DID estimates with state-year fixed effects were produced in response to reviewer suggestions and hence were not part of the prespecified analysis plan. All analyses were conducted between February 5 and October 28, 2025, using Stata, version 18 (StataCorp LLC). The threshold for significance was set at a 2-sided *P* < .05.

## Results

The final analytic sample included 170 604 county-month combinations (677 counties multiplied by 252 months) based on 11 479 922 singleton births (mean [SD] gestational age, 39.5 [0.8] weeks; 51.2% boys and 48.8% girls; mean [SD] birth weight, 3337.4 [172.8] g; highest mean [SD] maternal age proportions, 0.36 [0.13] aged 20-24 years and 0.27 [0.12] aged 25-29 years; mean [SD] maternal racial proportions, 0.14 [0.22] Black, 0.84 [0.22] White, and 0.02 [0.06] other) across 677 counties (408 CWF treated [60.3%] and 269 [39.7%] never treated) (Table). Overall, birth outcomes and maternal characteristics appeared broadly similar across treated and never-treated counties.

**Table.** Summary Statistics of the Main Outcome and Control Variables Across County Groups

Characteristic	Proportion, mean (SD)		
	All counties	Treated counties	Never-treated counties
**Newborn**			
Birth weight, g	3337.4 (172.8)	3333.3 (175.0)	3343.6 (169.1)
Low birth weight^a^	0.07 (0.07)	0.07 (0.07)	0.07 (0.07)
Gestational age, wk	39.5 (0.8)	39.5 (0.8)	39.5 (0.8)
Premature birth^b^	0.10 (0.10)	0.10 (0.10)	0.09 (0.10)
**Maternal**			
Race^c^			
Black	0.14 (0.22)	0.16 (0.22)	0.87 (0.21)
White	0.84 (0.22)	0.82 (0.22)	0.87 (0.21)
Other	0.02 (0.06)	0.02 (0.07)	0.02 (0.05)
Age group, y			
<20	0.20 (0.12)	0.21 (0.12)	0.18 (0.12)
20-24	0.36 (0.13)	0.36 (0.13)	0.36 (0.13)
25-29	0.27 (0.12)	0.26 (0.12)	0.28 (0.12)
30-34	0.12 (0.09)	0.12 (0.09)	0.13 (0.09)
35-39	0.04 (0.06)	0.04 (0.05)	0.04 (0.05)
>39	0.01 (0.03)	0.01 (0.03)	0.01 (0.03)
Birth order			
First child	0.39 (0.14)	0.40 (0.14)	0.39 (0.13)
Second child	0.31 (0.13)	0.31 (0.13)	0.31 (0.12)
Third child	0.15 (0.10)	0.15 (0.10)	0.16 (0.10)
Fourth child or higher	0.15 (0.13)	0.15 (0.14)	0.15 (0.12)
Counties, No.	677	408	269
Observations, No.	170 604	102 816	67 788

^a^ Low birth weight defined as a birth weight less than 2500 g.

^b^ Premature birth defined as gestational age 37 weeks or younger.

^c^ Race categorization of 1968 was used.

**Figure 1.**
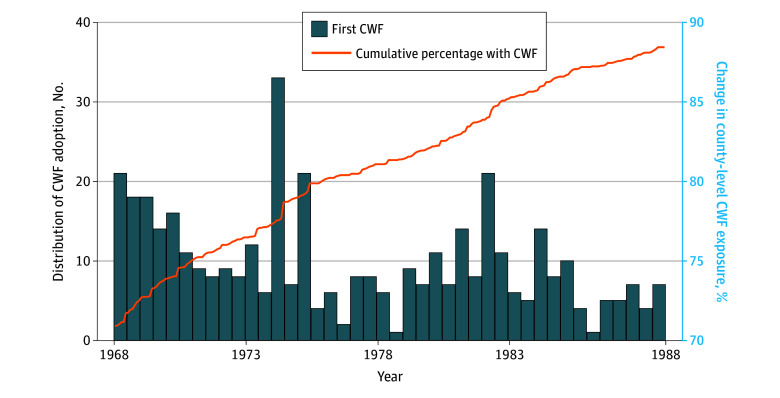
County-Level Community Water Fluoridation (CWF) Over the Study Period

Figure 1 illustrates the change in county-level CWF exposure over time, showing a steady increase throughout the study period. By the end of 1988, 2056 counties covered in the CWF data (88.4%) had adopted CWF, corresponding to 46.1% of the population. The figure also displays the distribution of CWF adoption years, confirming the staggered rollout across counties.

**Figure 2.**
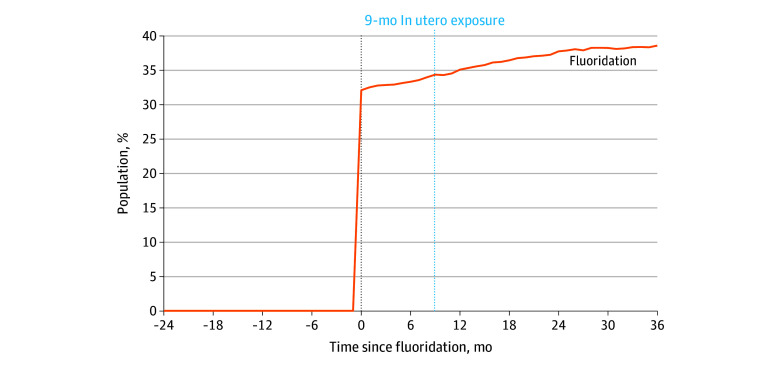
Cumulative Population With Fluoridated Water Before and After Initial Fluoridation Shown is the mean percentage of a county’s total population with access to fluoridated water (in months) since the initial fluoridation date (vertical dotted line at 0).

Figure 2 shows the change in population exposure to CWF following fluoridation. Because counties were often served by multiple water districts that may not have been universally fluoridated or may have begun fluoridation at different times, the increase in exposure did not reach 100% (ie, complete fluoridation of all water sources in a given county). Adoption of CWF led to a mean (SD) increase of 32.1 (27.5) percentage points in the proportion of the county population with fluoridated water.

**Figure 3.**
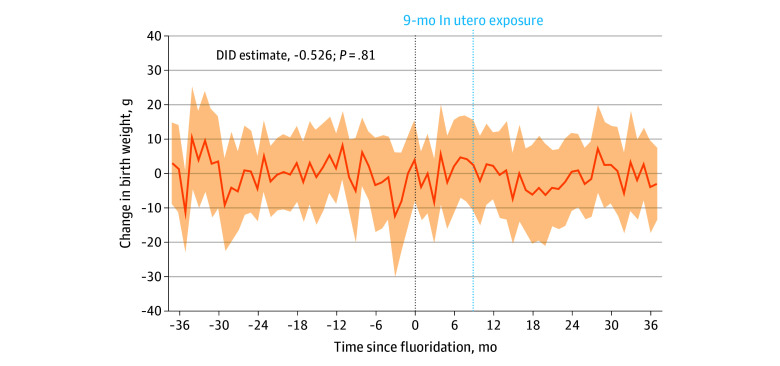
Estimated Association Between Community Water Fluoridation and Birth Weight Regression coefficients are shown with 95% CIs (shaded areas) from fixed-effects regressions of birth weight on 36 indicators for months preceding initial community water fluoridation (CWF), an indicator for the month of initial CWF, and 37 indicators for months following CWF. The month immediately before initial CWF (vertical dotted line at 0) served as the reference period. The difference-in-differences (DID) estimate corresponds to an equivalent regression specification that pooled months 9 through 20 after fluoridation (first year with full CWF exposure) into a single coefficient, with months −12 to −1 (pre-CWF year) serving as the reference period.

Figure 3 presents event-study estimates for birth weight. Event-study estimates showed no discernible pretreatment trends and no significant changes following CWF adoption, with estimates small in magnitude across all posttreatment periods, ranging from −8.44 g (95% CI, −20.41 to 3.53 g) to 7.20 g (95% CI, −5.45 to 19.85 g). Vertical lines denote the year of fluoridation and the point 9 months afterward, marking the first cohort with full prenatal exposure. The pretreatment coefficients were statistically indistinguishable from 0, with minor year-to-year fluctuations consistent with sampling variability. This absence of pretrends supports the internal validity of the design by suggesting that counties that adopted CWF and those that did not followed similar trends in birth weight outcomes prior to treatment. The posttreatment coefficients were also close to 0 and not statistically significant in the period immediately following CWF adoption and in all subsequent periods (DID estimate, −0.53; 95% CI, −4.75 to 3.70; *P* = .81). The range of the 95% CIs across posttreatment estimates was −21.2 to 20.3.

In sensitivity analyses, the event-study estimates replicated the analysis for the share of births classified as low birth weight (DID estimate, 0.15; 95% CI, −2.26 to 2.57; *P* = .91), gestational length (DID estimate, −0.01; 95% CI, −0.04 to 0.02; *P* = .34), and risk of prematurity (DID estimate, 0.88; 95% CI, −3.45 to 5.41; *P* = 0.70) (respectively, eFigures 1-3 in Supplement 1). Consistent with the birth weight results, we found no evidence of a pretrend or any other association between CWF and these outcomes. Reestimations using the sample of counties with more than 90% fluoridation coverage to address potential misclassification remained nonsignificant and similar in magnitude (DID estimate, 2.43; 95% CI, −6.15 to 11.00; *P* = .58) (eFigure 4 in Supplement 1). We also estimated a model using state-by-year fixed effects, which flexibly accounted for differential, nonlinear secular trends across states, and found no influence on our main results (eFigure 5-8 in Supplement 1). To explore changes in population in response to CWF, we specified the dependent variable as the number of births per county-month and reestimated our event-study models (eFigure 9 in Supplement 1). No association between CWF and the number of births was found, but some evidence of trending over the entire period was observed. Including state-by-year-by-month fixed effects removed the potential trending (eFigure 10 in Supplement 1).

## Discussion

After observing that communities with higher naturally occurring fluoride levels in their water supplies had significantly lower rates of tooth decay, a major public health experiment was launched in 1945 in Grand Rapids, Michigan, in which fluoride was added to the municipal water system, and the nearby city of Muskegon served as a control. By 1950, dental caries in children in Grand Rapids had declined by 60%.^19^ Similar experiments in other US and international cities produced comparable results, prompting a rapid expansion of CWF across the US from just 3.3% of the population in 1951 to 63% by 2018. More recent evidence has suggested smaller effects, typically in the range of 25% to 35%,^20^ with the decline largely attributable to the widespread availability of other fluoride-based preventive measures, including fluoridated toothpaste, fluoride varnishes, and dental sealants, which have reduced the marginal impact of fluoridated drinking water.

There are many explored adverse effects from fluoride ingestion. Excessive intake of fluoride may cause fluorosis, a cosmetic discoloration of the teeth, though this typically occurs at levels beyond which CWF is adjusted. More serious, though more disputed, is the purported association between fluoride intake and other health outcomes, notably bone cancer in children (osteosarcoma),^21^ although laboratory and epidemiologic evidence do not support this association.^22^ A growing body of evidence has examined whether fluoride exposure is associated with IQ,^23,24,25,26,27^ again with inconclusive results.

This cohort study investigated the association between CWF exposure and birth weight and gestational length using an event-study DID design that exploited the staggered rollout of CWF over time. In all analyses, no statistically significant association was found between the two. Furthermore, the estimated coefficients were small in magnitude. Even under a liberal interpretation that focused on the lower bound of the 95% CI across posttreatment estimates, the maximum estimated coefficient across all months was −21.2 g, which is less than 1% of the mean birth weight for the control counties (3343.6 g). Moreover, the posttreatment coefficients were similar in magnitude to pretreatment coefficients, which were only included to assess the internal validity of the model. Together, these findings suggest that any coefficient estimate was not only statistically undetectable but also clinically not significant.

These results stand in contrast to previous research on fluoride exposure and birth weight, which either explored maternal urinary fluoride levels during pregnancy^7,8,28^ or exposure to CWF,^29,30^ producing considerable variation in findings, both positive and negative. We believe that the key reason for this divergence from previous findings lies in the research design. Earlier studies relied on cross-sectional analyses, which are susceptible to confounding. In contrast, our event-study DID framework, which exploited variation in the timing of CWF adoption across counties, substantially mitigated the risk of confounding and, as such, we believe improved the credibility of our design.

Although we lacked a randomized experiment, our estimates may be interpreted as causal under the assumption that the timing of CWF adoption is not systematically correlated with other determinants of newborn health. Several factors support this assumption. For example, cities within the same state adopted fluoridation at markedly different times, such as Nashville (1953) and Memphis (1970) in Tennessee and Cleveland (1956) and Columbus (1973) in Ohio, despite similar access to public health guidance.^13^ This variation appears idiosyncratic and unlikely to be systematically associated with changes in perinatal health. More importantly, our staggered DID approach leveraged within-municipality changes over time, not across-municipality comparisons, further limiting the potential for confounding.

Empirically, several of our findings reinforce this interpretation. We found no evidence of differential pretrends in birth weight between fluoridated and nonfluoridated counties, which supports the parallel trends assumption required for causal inference in DID models. Our results remained stable after controlling for state-specific time trends, which allowed for differential secular patterns across states. We also found no evidence to support compositional changes in response to CWF as measured by the number of births.

### Limitations

Our study had several limitations. A key distinction of our study is that by measuring fluoride exposure at the community rather than individual level, we provided an estimate of a group-level intention to treat, which is distinct from the individual-level treatment-on-the-treated estimate captured by biomarker-based studies.^7^ However, community-level measures of CWF introduce exposure misclassification because multiple water districts can serve residents in a county, which could attenuate treatment estimates. (The same misclassification issue does not arise, however, for our outcome variables because we knew the county of birth.) Since the change in CWF status ranged from 0% to 100%, we reestimated our models using the sample of counties with more than 90% fluoridation coverage to address potential misclassification errors. Our results remained statistically nonsignificant and similar in magnitude (eFigure 4 in Supplement 1), indicating that the threat from misclassification was minimal in this setting.

While our research design limited the threat of unobserved confounding, we could not fully rule it out in this observational analysis. For example, other trends occurring over the same period, such as environmental regulations that impacted water and air quality, including the Safe Drinking Water Act and Clean Air Act, may have impacted infant health. Such policies were implemented at the national or state level, as opposed to the water district level for CWF, thereby limiting their threat. As evidence supporting this claim, we estimated a model with state-by-year fixed effects, which flexibly accounted for differential, nonlinear secular trends across states, and found that these trends did not influence our main results (eFigures 5-8 in Supplement 1). While we cannot definitively rule out confounding from such policies, we believe the threat to be low.

A more important concern is that water districts may have implemented other treatment technologies, such as adding disinfectants and corrosion control agents and removing impurities, such as pollutants, at the same time they adopted CWF. For one of these factors to confound estimates, it must occur at the same time an area adopts CWF. Absent further data availability, we were unable to directly assess whether this occurred. Given that we reported null estimates across all outcomes, we view the potential for confounding as low: Any coincidental treatment changes would need to precisely offset the estimates for CWF to yield 0 net estimates.

Another potential concern related to changes in the population in response to CWF. To explore this concern, we specified the dependent variable as the number of births per county-month and reestimated our event study models. As shown in eFigures 9 and 10 in Supplement 1, we did not find a statistically significant association between CWF and the number of births, though with some visual evidence of trending occurring over the entire period. Including state-by-year-by-month fixed effects, however, removed the potential trending. This result suggests that population changes were unlikely in this setting.

## Conclusions

This cohort study found that CWF is not associated with infant health as measured by birth weight, contributing to ongoing evaluations of the safety of CWF, particularly with regard to potential adverse effects during pregnancy. Our findings contribute to the broader discussion of potential adverse effects of fluoride exposure and highlight the importance of using more rigorous empirical strategies when evaluating population-level interventions. Although we did not directly assess the debated association between fluoride and cognitive outcomes, our findings raise broader questions about the reliability of associational evidence. If the observed associations between CWF and birth weight dissipate under more rigorous statistical designs, it would be worth considering whether similar patterns may hold for CWF and neurodevelopmental outcomes. Future work using stronger research designs would be essential in evaluating these concerns more definitively.

## Notes

Supplement 1.
eMethods.

eReferences
**eFigure 1.** Estimated Association Between Community Water Fluoridation and Low Birth Weight **eFigure 2.** Estimated Association Between Community Water Fluoridation and Gestational Length **eFigure 3.** Estimated Association Between Community Water Fluoridation and Prematurity **eFigure 4.** Estimated Association Between Community Water Fluoridation and Birth Weight, Including Only More Than 90%-Treated Counties **eFigure 5.** Estimated Association Between Community Water Fluoridation and Birth Weight, Allowing for State-Specific Time Trends **eFigure 6.** Estimated Association Between Community Water Fluoridation and Low Birth Weight, Allowing for State-Specific Time Trends **eFigure 7.** Estimated Association Between Community Water Fluoridation and Gestational Length, Allowing for State-Specific Time Trends **eFigure 8.** Estimated Association Between Community Water Fluoridation and Prematurity, Allowing for State-Specific Time Trends **eFigure 9.** Estimated Association Between Community Water Fluoridation and Birth Rate **eFigure 10.** Estimated Association Between Community Water Fluoridation and Birth Rate, Including State-by-Year-by-Month Fixed Effects

Supplement 2.
Data Sharing Statement
