# Supplementary material for: Community Water Fluoridation and Birth Outcomes
Source: JAMA Netw Open. 2026 Jan 20;9(1):e2554686. doi: 10.1001/jamanetworkopen.2025.54686 (PMC12820739; doi:10.1001/jamanetworkopen.2025.54686)
Supplement: Supplement 1. — eMethods. eReferences eFigure 1. Estimated Association Between Community Water Fluoridation and Low Birth Weight eFigure 2. Estimated Association Between Community Water Fluoridation and Gestational Length eFigure 3. Estimated Association Between Community Water Fluoridation and Prematurity eFigure 4. Estimated Association Between Community Water Fluoridation and Birth Weight, Including Only More Than 90%-Treated Counties eFigure 5. Estimated Association Between Community Water Fluoridation and Birth Weight, Allowing for State-Specific Time Trends eFigure 6. Estimated Association Between Community Water Fluoridation and Low Birth Weight, Allowing for State-Specific Time Trends eFigure 7. Estimated Association Between Community Water Fluoridation and Gestational Length, Allowing for State-Specific Time Trends eFigure 8. Estimated Association Between Community Water Fluoridation and Prematurity, Allowing for State-Specific Time Trends eFigure 9. Estimated Association Between Community Water Fluoridation and Birth Rate eFigure 10. Estimated Association Between Community Water Fluoridation and Birth Rate, Including State-by-Year-by-Month Fixed Effects [file jamanetwopen-e2554686-s001.pdf]

Supplemental Online Content

Krebs B, Simon L, Schwandt H, Burn S, Neidell M. Community water fluoridation and birth outcomes. *JAMA Netw Open*. 2026;9(1):e2554686. doi:10.1001/jamanetworkopen.2025.54686

eMethods
eReferences
eFigure 1. Estimated Association Between Community Water Fluoridation and Low Birth Weight
eFigure 2. Estimated Association Between Community Water Fluoridation and Gestational Age
eFigure 3. Estimated Association Between Community Water Fluoridation and Prematurity
eFigure 4. Estimated Association Between Community Water Fluoridation and Birth Weight, Including Only More Than 90%-Treated Counties
eFigure 5. Estimated Association Between Community Water Fluoridation and Birth Weight, Allowing for State-Specific Time Trends
eFigure 6. Estimated Association Between Community Water Fluoridation and Low Birth Weight, Allowing for State-Specific Time Trends
eFigure 7. Estimated Association Between Community Water Fluoridation and Gestational Age, Allowing for State-Specific Time Trends
eFigure 8. Estimated Association Between Community Water Fluoridation and Prematurity, Allowing for State-Specific Time Trends
eFigure 9. Estimated Association Between Community Water Fluoridation and Birth Rate
eFigure 10. Estimated Association Between Community Water Fluoridation and Birth Rate, Including State-by-Year-by-Month Fixed Effects

This supplemental material has been provided by the authors to give readers additional information about their work.

© 2026 Krebs B et al. *JAMA Network Open*.

eMethods

To implement the difference-in-difference event study design, we follow [1] to estimate the following equation:

$$Y_{jt} = \sum_{k=-37}^{-2} \gamma_k \cdot 1\{K_{jt} = k\} + \sum_{k=0}^{37} \beta_k \cdot 1\{K_{jt} = k\} + \partial \cdot X_{jt} + \sigma_t + \alpha_j + \epsilon_{jt}$$

where j indexes county and t time, and Y_{jt} represents our primary outcome of interest: county-level birth weight. As secondary outcomes, we explore the number of births that are low birth weight (< 2500g), gestational length in weeks, and the number of premature births (gestation < 37 weeks). For birth weight and gestational length, we estimate a linear regression model. For the number of births that are low birth weight or premature, we estimate a Poisson quasi-maximum likelihood model with the number of births as the offset.

Since these models are estimated at the county level using a group-level treatment, we estimate the impact from community water fluoridation rather than the impact from individual exposure to fluoride, thus reflecting an intent to treat.

The following control variables are included: X_{jt} are county demographic controls shown in Table 1 to account for maternal characteristics, σ_t are year-by-month fixed effects to account for seasonal patterns separately by year, and α_j are county fixed effects to limit comparisons to changes in the outcome over time within each county. Observations are weighted by the number of births in the county-month cell, and the error term (ε_{jt}) is clustered at the county level to account for serial correlation.

Our main object of interest is γ_k and β_k, the vector of event-study coefficients from 36 months before and after changes in CWF status. Importantly, these are distinct from the time fixed effects (σ_t), which capture overall temporal variation across the study period. Specifically, the $1\{K_{jt=k}\}$ is an indicator function used to denote whether the time period is *k* months before or after CWF adoption. The pre-

© 2026 Krebs B et al. *JAMA Network Open*.

treatment coefficients (γ_k) test for pre-existing trends as an assessment of internal validity of the model. The post-treatment coefficients (β_k) assess if CWF is negatively associated with birth weight. If CWF impedes fetal development, then we expect negative coefficients for the post-treatment coefficients. We report test results for each coefficient separately, and also provide the p-value from an F-test of joint significance separately for all pre-treatment coefficients and for all post-treatment coefficients. Given the degree of CWF exposure varies in the first 9 months after CWF adoption, we also focus on the post-treatment coefficients beginning with β_9, where newborns have experienced CWF exposure for the entire prenatal period. We use the month before the change in CWF status as the reference category (t = -1) and interpret all estimates as relative to that month. (Note that we apply -37 to all months before -36 and 37 to all months after 36; and never-treated counties are assigned -37.)

This model differs from a traditional two-period difference-in-differences (DiD) model in two important ways. First, exploiting the staggered adoption of CWF over time allows for greater control of general time trends that affect all counties. For example, if County A adopts CWF in 1980 and County B in 1985, the within-county difference for County A compares outcomes before and after 1980, while for County B it compares before and after 1985. At each point in time, counties that are more than 36 months before or more than 36 months after adoption serve as controls for those that are within the 72-month window surrounding adoption. Thus, in 1980, County A (treated) is compared with County B (not yet treated), and in 1985 the roles reverse. In this sense, it is a weighted average of many individual DiDs [2].

Second, we allow for dynamic treatment effects via the event study design. That is, instead of one post-treatment variable, we have a series of post-treatment variables indicated by months since CWF adoption. This approach allows for a flexible treatment effect to vary over time.

We also produce a more traditional DiD estimates using the method described in [3]. Specifically, we define two pre-treatment periods (−37 to −13 months and −12 to −1 months) and three

© 2026 Krebs B et al. *JAMA Network Open*.

post-treatment periods (0 to 8 months, 9 to 20 months, and 21 to 37 months). The full year prior to CWF adoption (−12 to −1 months) serves as the reference category. We report the estimate for the 9–20 month period as the DiD estimate, which corresponds to the interval in which all newborns were exposed to CWF throughout the entire prenatal period. This still exploits the staggered rollout of CWF, but limits the time frame to immediately before and after CWF. These estimates are reported for all outcomes within each figure.

eReferences

[1] Schmidheiny, Kurt and Sebastian Siegloch. 2023. "On event studies and distributed-lags in two-way fixed effects models: Identification, equivalence, and generalization." *Journal of Applied Econometrics*, 2023, 38 (5), 695-713.

[2] Goodman-Bacon, Andrew. 2021. "Difference-in-Differences with Variation in Treatment Timing." *Journal of Econometrics* 225 (2): 254–77.

[3] Miller, Douglas L. 2023. "An Introductory Guide to Event Study Models." *Journal of Economic Perspectives* 37 (2): 203–30.

© 2026 Krebs B et al. *JAMA Network Open*.

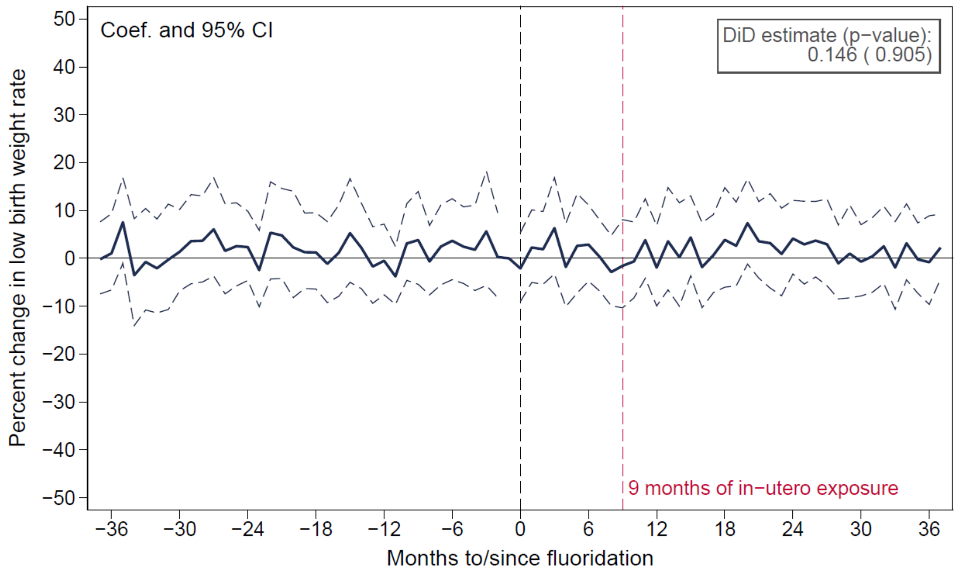

eFigure 1. Estimated Association Between Community Water Fluoridation and Low Birth Weight (birth weight < 2500 grams). **Notes:** Regression coefficients (solid line) and 95% confidence intervals (dashed lines) from Poisson pseudo maximum likelihood fixed-effects regressions using the number of low-birth weight births as the dependent variable and the log number of births as the offset. See notes to Figure 3 for further details. The DiD estimate corresponds to an equivalent regression specification that pools months 9–20 after fluoridation (first year with full CWF exposure) into a single coefficient, with months −12 to −1 (pre-CWF year) serving as the reference period.

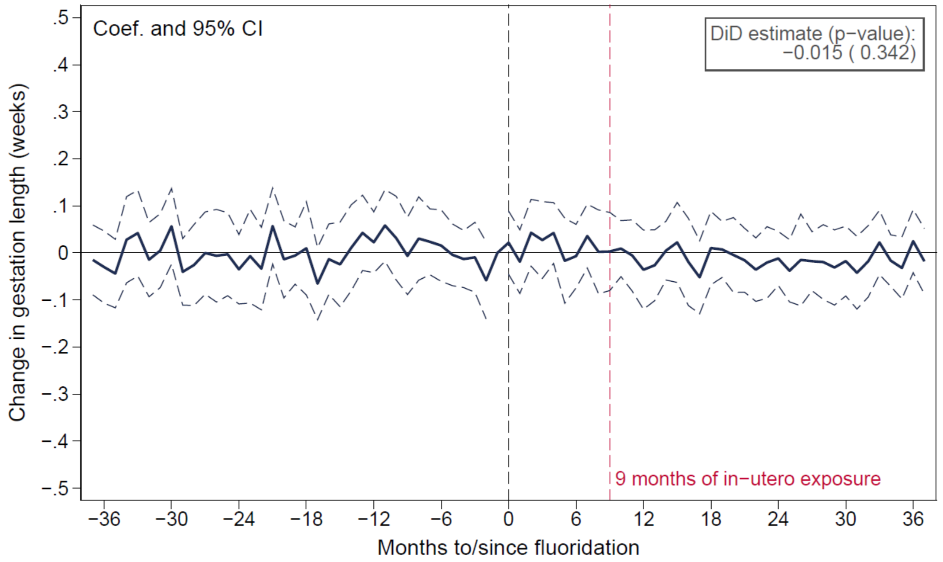

eFigure 2. Estimated Association Between Community Water Fluoridation and Gestational Age (in weeks). **Notes:** Regression coefficients (solid line) and 95% confidence intervals (dashed lines) from fixed-effects regressions using gestational length as the dependent variable. See notes to Figure 3 for further details. The DiD estimate corresponds to an equivalent regression specification that pools months 9–20 after fluoridation (first year with full CWF exposure) into a single coefficient, with months −12 to −1 (pre-CWF year) serving as the reference period.

© 2026 Krebs B et al. *JAMA Network Open*.

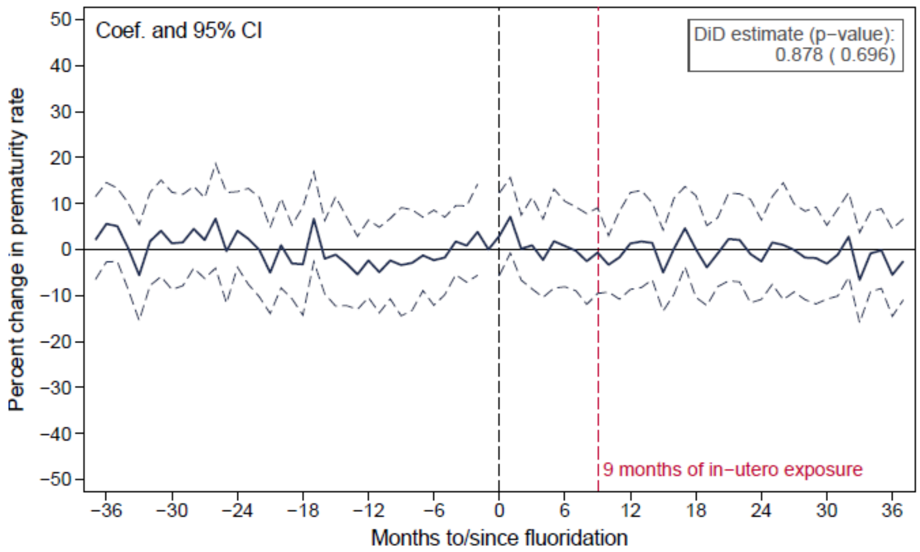

eFigure 3. Estimated Association Between Community Water Fluoridation and Prematurity (gestational length ≤ 37 weeks). **Notes:** Regression coefficients (solid line) and 95% confidence intervals (dashed lines) from Poisson pseudo maximum likelihood fixed-effects regressions using the number of premature births as the dependent variable and the log number of births as the offset. See notes to Figure 3 for further details. The DiD estimate corresponds to an equivalent regression specification that pools months 9–20 after fluoridation (first year with full CWF exposure) into a single coefficient, with months −12 to −1 (pre-CWF year) serving as the reference period.

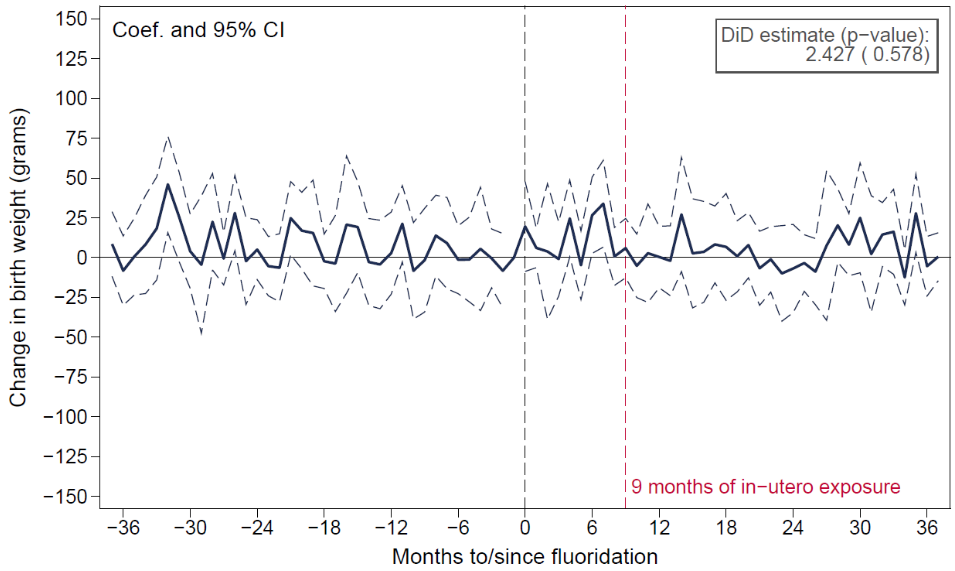

eFigure 4. Estimated Association Between Community Water Fluoridation and Birth Weight (in grams), Including Only More Than 90%-Treated Counties. **Notes:** Regression coefficients (solid line) and 95% confidence intervals (dashed lines) from fixed-effects regressions using birth weight as the dependent variable. The regression sample only includes > 90%-treated counties and never-treated counties. See notes to Figure 3 for further details. The DiD estimate corresponds to an equivalent regression specification that pools months 9–20 after fluoridation (first year with full CWF exposure) into a single coefficient, with months −12 to −1 (pre-CWF year) serving as the reference period.

© 2026 Krebs B et al. *JAMA Network Open*.

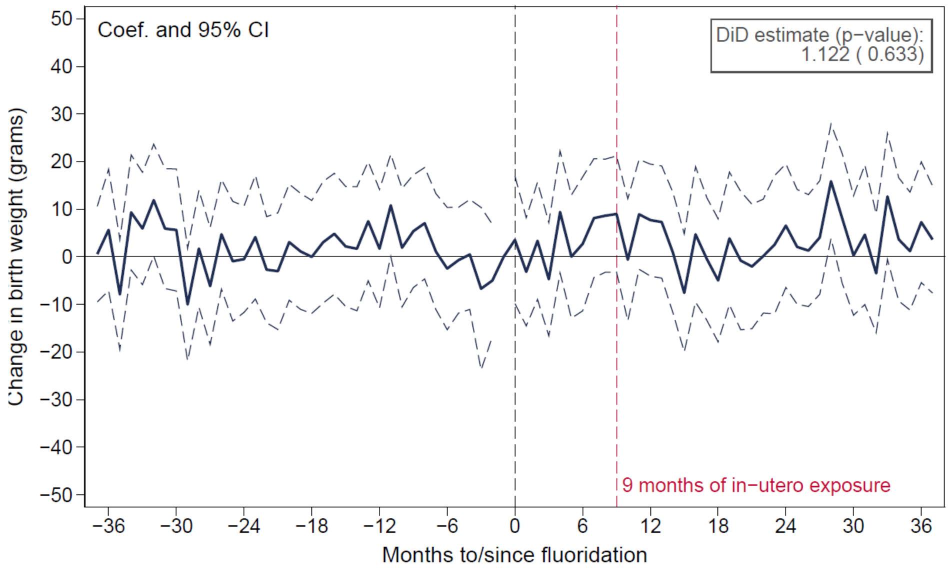

eFigure 5. Estimated Association Between Community Water Fluoridation and Birth Weight (in grams), Allowing for State-Specific Time Trends. **Notes:** Regression coefficients (solid line) and 95% confidence intervals (dashed lines) from fixed-effects regressions using birth weight as the dependent variable. The model includes state-by-month-by-year fixed effects instead of month-by-year fixed effects. See notes to Figure 3 for further details. The DiD estimate corresponds to an equivalent regression specification that pools months 9–20 after fluoridation (first year with full CWF exposure) into a single coefficient, with months −12 to −1 (pre-CWF year) serving as the reference period.

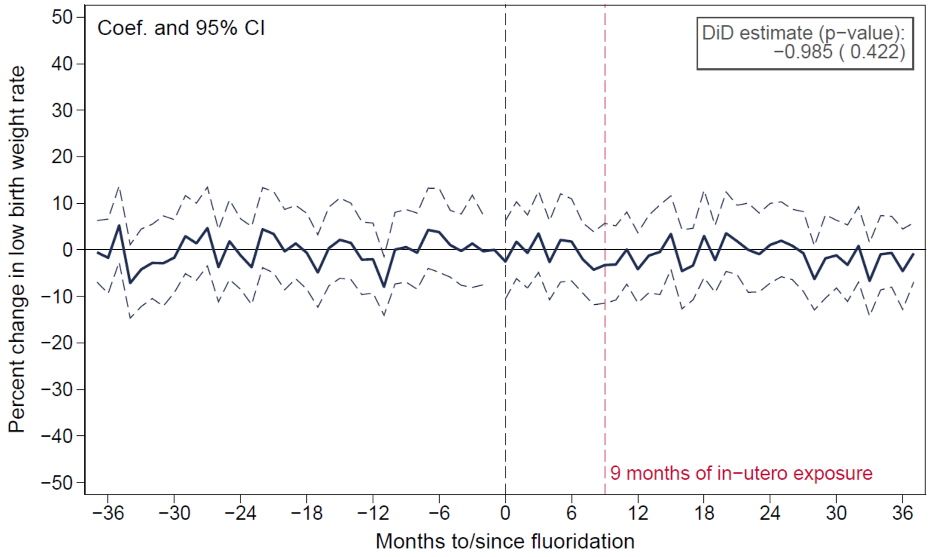

eFigure 6. Estimated Association Between Community Water Fluoridation and Low Birth Weight (birth weight <2500 grams), Allowing for State-Specific Time Trends. **Notes:** Regression coefficients (solid line) and 95% confidence intervals (dashed lines) from Poisson pseudo maximum likelihood fixed-effects regressions using number of low-birth weight births as the dependent variable and the log number of births as the offset. The model includes state-by-month-by-year fixed effects instead of month-by-year fixed effects. See notes to Figure 3 for further details. The DiD estimate corresponds to an equivalent regression specification that pools months 9–20 after fluoridation (first year with full CWF exposure) into a single coefficient, with months −12 to −1 (pre-CWF year) serving as the reference period.

© 2026 Krebs B et al. *JAMA Network Open*.

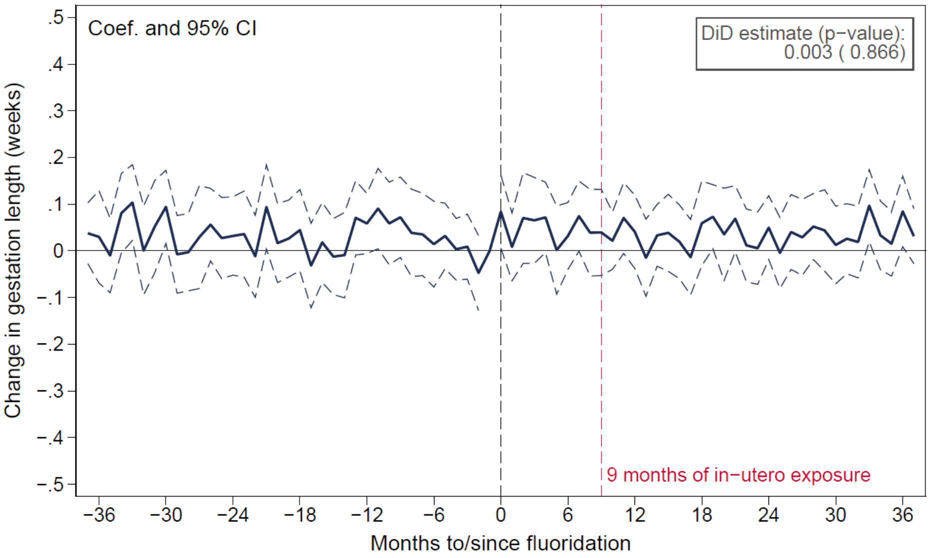

eFigure 7. Estimated Association Between Community Water Fluoridation and Gestational Age (in weeks), Allowing for State-Specific Time Trends. **Notes:** Regression coefficients (solid line) and 95% confidence intervals (dashed lines) from linear fixed-effects regressions using gestational length as the dependent variable. The model includes state-by-month-by-year fixed effects instead of month-by-year fixed effects. See notes to Figure 3 for further details. The DiD estimate corresponds to an equivalent regression specification that pools months 9–20 after fluoridation (first year with full CWF exposure) into a single coefficient, with months −12 to −1 (pre-CWF year) serving as the reference period.

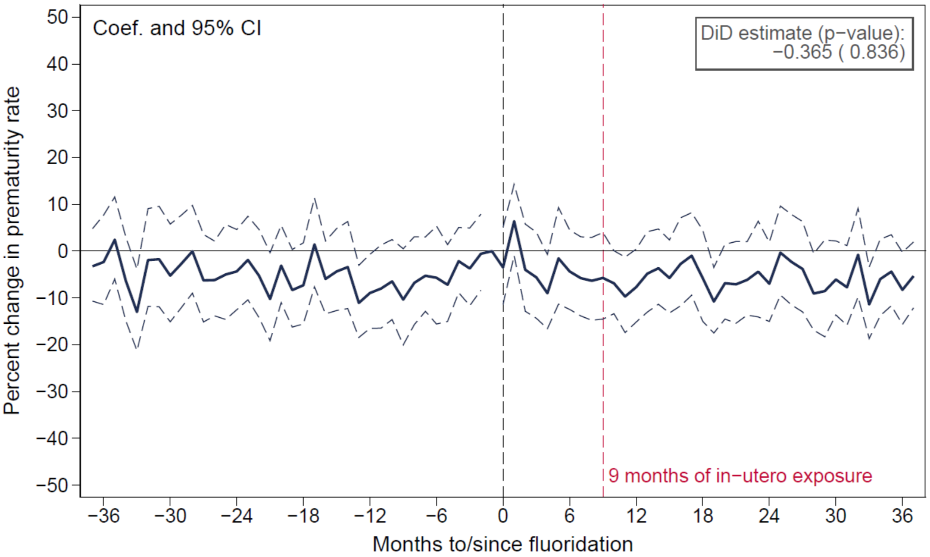

eFigure 8. Estimated Association Between Community Water Fluoridation and Prematurity (gestational length ≤ 37 weeks), Allowing for State-Specific Time Trends. **Notes:** Regression coefficients (solid line) and 95% confidence intervals (dashed lines) from Poisson pseudo maximum likelihood fixed-effects regressions using the number of premature births as the dependent variable and the log number of births as the offset. The model includes state-by-month-by-year fixed effects instead of month-by-year fixed effects. See notes to Figure 3 for further details. The DiD estimate corresponds to an equivalent regression specification that pools months 9–20 after fluoridation (first year with full CWF exposure) into a single coefficient, with months −12 to −1 (pre-CWF year) serving as the reference period.

© 2026 Krebs B et al. *JAMA Network Open*.

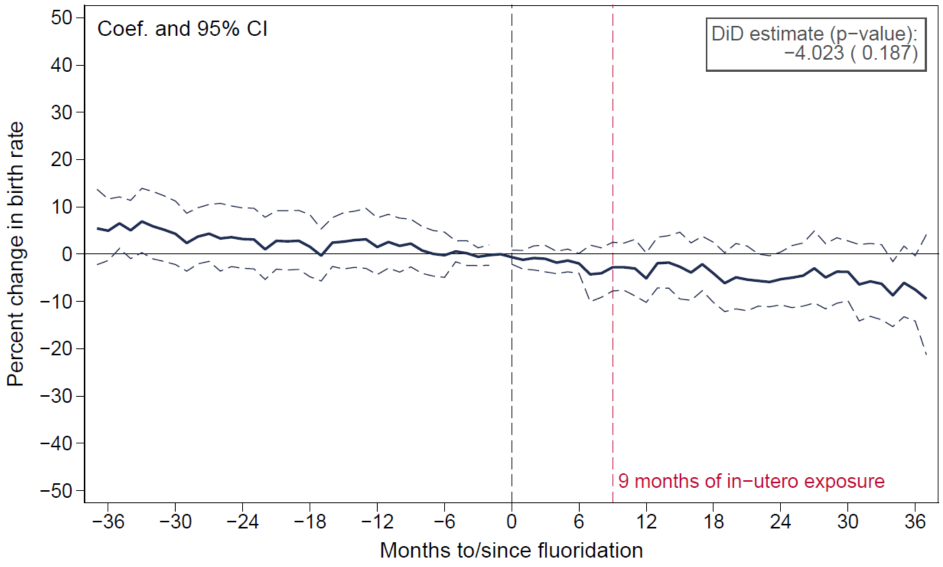

eFigure 9. Estimated Association Between Community Water Fluoridation and Birth Rate. **Notes:** Regression coefficients (solid line) and 95% confidence intervals (dashed lines) from Poisson pseudo maximum likelihood fixed-effects regressions using the number of births as the dependent variable and the log number of females between 15 and 44 years old as the offset. See notes to Figure 3 for further details. The DiD estimate corresponds to an equivalent regression specification that pools months 9–20 after fluoridation (first year with full CWF exposure) into a single coefficient, with months −12 to −1 (pre-CWF year) serving as the reference period.

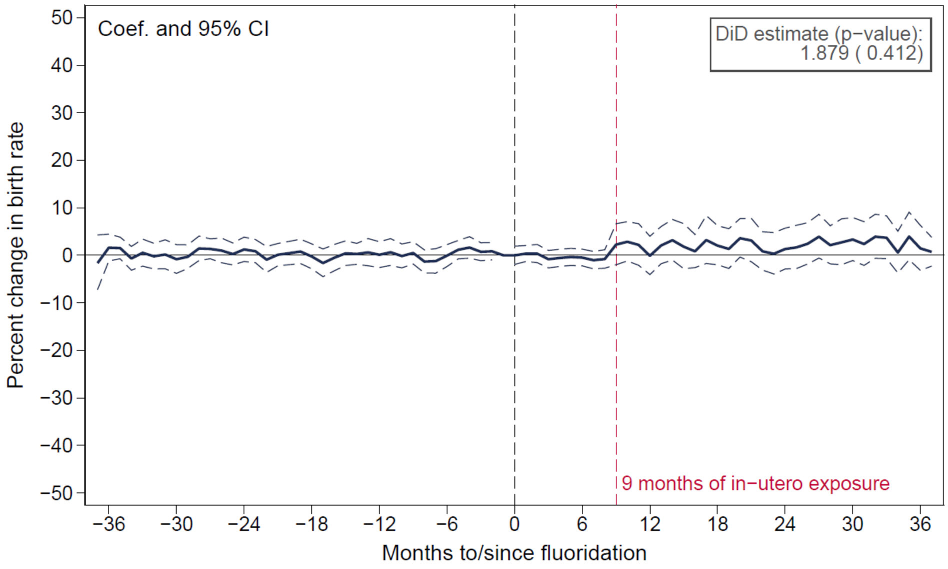

eFigure 10. Estimated Association Between Community Water Fluoridation and Birth Rate, Including State-by-Year-by-Month Fixed Effects. **Notes:** Regression coefficients (solid line) and 95% confidence intervals (dashed lines) from Poisson pseudo maximum likelihood fixed-effects regressions using the number of births as the dependent variable and the log number of females between 15 and 44 years old as the offset. The model includes state-by-month-by-year fixed effects instead of month-by-year fixed effects. See notes to Figure 3 for further details. The DiD estimate corresponds to an equivalent regression specification that pools months 9–20 after fluoridation (first year with full CWF exposure) into a single coefficient, with months −12 to −1 (pre-CWF year) serving as the reference period.

© 2026 Krebs B et al. *JAMA Network Open*.
