# Supplementary material for: Community Water Fluoridation and Birth Outcomes
Source: JAMA Netw Open. 2026 Jan 20;9(1):e2554686. doi: 10.1001/jamanetworkopen.2025.54686 (PMC12820739; doi:10.1001/jamanetworkopen.2025.54686)
Supplement: Supplement 2. — Data Sharing Statement [file jamanetwopen-e2554686-s002.pdf]

Data Sharing Statement

Data
Data available: Yes
Data types: Data (not involving human participants), Data dictionary
How to access data: The birth outcome data we use is a restricted use version that requires approval. We will provide information on how to obtain the data and all code for processing that data. The fluoridation exposure data will be posted online
When available: With publication

Supporting Documents
Document types: Statistical/analytic code, Other (please specify)
Additional Information: We will provide all processing and statistical analysis code
How to access documents: On the website of the corresponding author
When available: With publication

Additional Information
Who can access the data: anyone requesting the data
Types of analyses: for any purpose
Mechanisms of data availability: freely available to all
